# Congenital Fusion of Spermatic Cords With Unilateral Seminal Vesicle Agenesis and Ipsilateral Varicocele in an Adult Male With Oligospermia: A Report of a Rare Case

**DOI:** 10.7759/cureus.89789

**Published:** 2025-08-11

**Authors:** Jad Kabbara, Mohannad Qasim, Moustafa Moussally

**Affiliations:** 1 Anesthesiology, Lake Erie College of Osteopathic Medicine, Westlake, USA; 2 Orthopedic Surgery, Lake Erie College of Osteopathic Medicine, Orland Park, USA; 3 General Surgery, American University of Beirut Medical Center, Beirut, LBN

**Keywords:** congenital birth defect, infertility, oligospermia, seminal vesicle agenesis, spermatic cord, varicocele ligation

## Abstract

Congenital anomalies of the male reproductive tract are significantly rare and are often discovered incidentally during infertility workups. This report identifies a rare case of a young male with oligospermia who is found to have congenital fusion of the spermatic cords, left unilateral seminal vesicle agenesis, as well as a left grade III varicocele. Workup established a lack of cystic fibrosis transmembrane conductance regulator (CFTR) mutation or urinary abnormalities usually associated with male anatomical reproductive alterations. A unilateral seminal vesicle agenesis is exceedingly rare and may lead to both obstructive and nonobstructive subfertility.

## Introduction

Congenital anomalies of the male genitourinary (GU) tract are significant causes of male infertility and can often appear with very few symptoms or incidentally, during imaging and surgical exploration. Among these anomalies is seminal vesicle agenesis, a rare condition typically associated with congenital absence of the vas deferens, which may be linked to a cystic fibrosis transmembrane conductance regulator (CFTR) gene mutation [1,2]. Among the rarest of the spermatic cord anomalies are fusions or aberrant pathways, which are extremely scarce and are very limited in the literature [3].

In males, the mesonephric (Wolffian) duct develops into several key components of the reproductive system under the influence of testosterone. These structures include the epididymis, which stores and matures sperm; the vas deferens (ductus deferens), which transports sperm from the epididymis to the ejaculatory duct; the seminal vesicles, which contribute fluid to semen; and the ejaculatory ducts, which deliver sperm and seminal fluid into the urethra. The kidneys and ureters are also derived from the mesonephric duct. This development occurs during embryogenesis and is critical for the formation of the male internal genital tract.

A more common GU tract anomaly that is seen is varicoceles, which present in up to 15% of the general male population and in 19%-41% of men who present with primary infertility [4]. Varicoceles are the most common cause of male infertility worldwide [4]. Although rarely associated with internal anomalies of the reproductive tract, inguinal hernia and undescended testes may be associated with this problem due to multifactorial issues. 

We present a highly unusual case of a young male who exhibited left unilateral seminal vesicle agenesis, fusion of the spermatic cord, right inguinal hernia, as well as a grade III varicocele, which were all discovered due to his unusual oligospermia. All structures that arise from the mesonephric duct in males were present in this patient, besides the left seminal vesicle. The operative findings and imaging provide insight into the clinical and embryological correlations.

## Case presentation

We present the case of an unmarried 25-year-old male, a regular smoker, who was referred to the Urology clinic by his general practitioner for evaluation of a dull, sudden-onset scrotal pain that had persisted for several days. He denied any associated lower urinary tract symptoms. Physical examination revealed a normal, circumcised phallus, with both testes intrascrotal and normal in size, consistency, and lie. Both epididymides were present, with a cystic mass noted on the left epididymal head; no palpable nodules were detected. A grade III varicocele was present on the left side. The spermatic cord was palpable on the right but not on the right. A right type 3a inguinal hernia was present. Spermogram showed oligospermia with a sperm count of 4.8 million/cc (normal: 33-46 million/cc); all other parameters within the spermogram were within normal range. Complete blood count (CBC), basic metabolic panel (BMP), lipid studies, testosterone levels, and urinalysis were all normal. 

**Figure 1:**
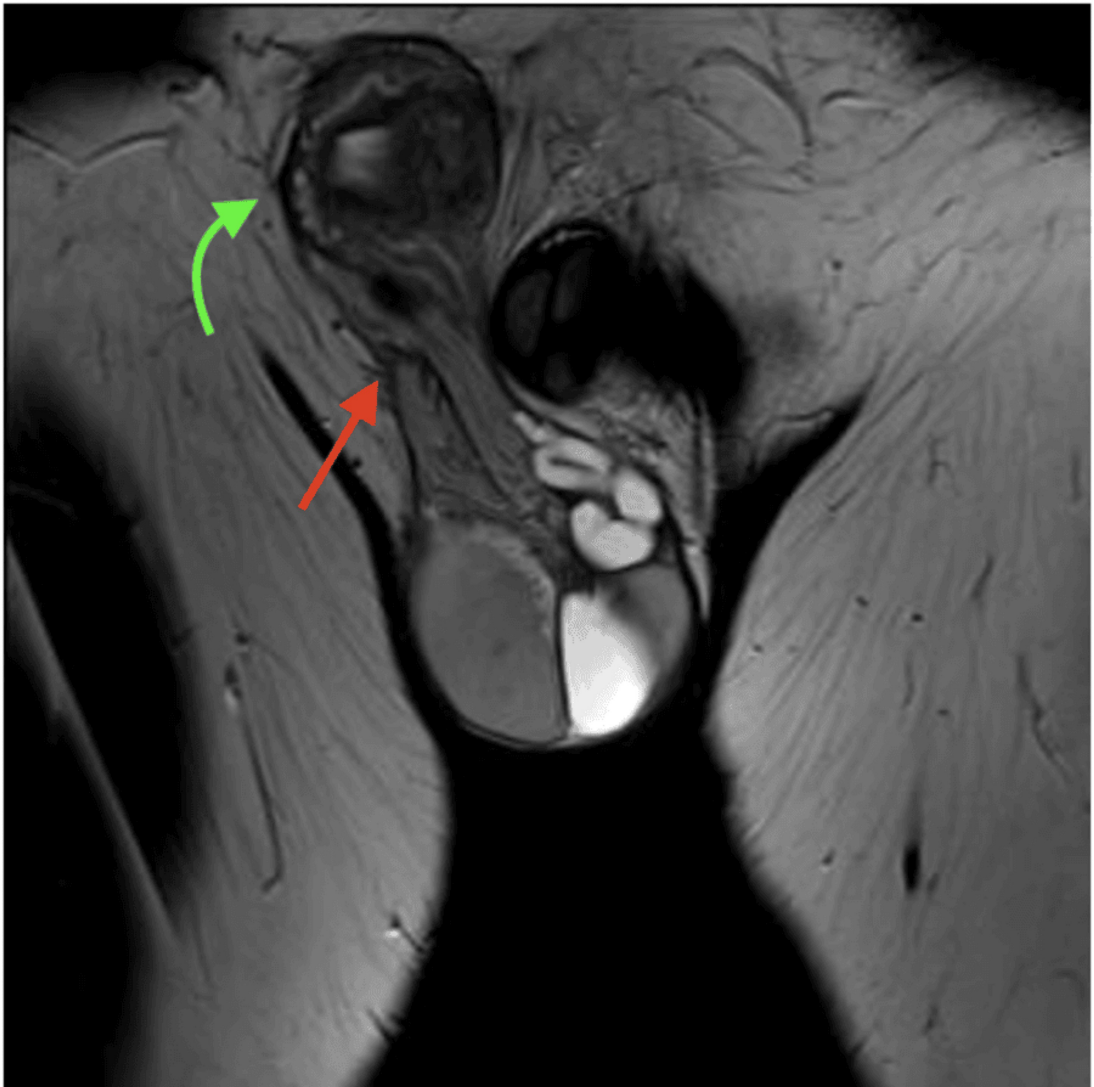
Coronal MRI image demonstrating right and left spermatic cord fusion. The red arrow indicates the fusion of the right and left spermatic cords, while the green arrow highlights a soft tissue structure at the site of the right spermatic cord near the inguinal region.

A 1.5T magnetic resonance imaging (MRI) of the pelvis revealed the absence of a left seminal vesicle with no left spermatic cord crossing the left inguinal area. Additionally, a 2.8 cm cystic structure with homogeneous, clear fluid is noted in the left scrotum with tubular structures that appear to arise from its superior aspect and extend toward the contralateral right spermatic cord. These tubular structures appear cystic in nature, demonstrating hyperintensity on T2-weighted images and hypointensity on T1-weighted images. MRI revealed fusion of the right and left spermatic cords (Figure 1), both directed into the right inguinal canal, with a single vas deferens originating from the right testis and connecting to a single right seminal vesicle.

When correlating both the ultrasound and MRI findings, the verdict was a complex congenital anomaly consisting of the absence of the left spermatic cord in the inguinal region, probably atretic or agenetic in this area, with continuation of the left cord along with the contralateral right one in the right inguinal area. There was agenesis of the left seminal vesicle, with a small dysgenetic left testicle associated with a grade III left varicocele and dilated left paratesticular tubular structures containing sperm.

For the sake of completion of radiological studies, a non-enhanced computed tomography scan of the abdomen and pelvis was performed. It showed normal size and position of both kidneys, normal bilateral ureters, a significantly dilated right vas deferens, and a normal right spermatic cord extending from the epididymis to its junction with the ejaculatory duct. The left spermatic cord originated from the small left testicle and crossed over to the contralateral side, with agenesis of the left seminal vesicle.

Owing to the absence of a seminal vesicle, a possible diagnosis of cystic fibrosis was considered; however, PCR amplification of a blood sample followed by sequence-based analysis was performed to screen for mutations in the CFTR gene responsible for cystic fibrosis. The result came out negative for any pathogenic mutation. 

Following those findings and the suboptimal sperm parameters, he underwent left internal spermatic vein ligation utilizing a transcrotal approach, and open exploration of the right inguinal canal, revealing the presence of two joining spermatic cords with the presence of two vas deferens originating from their respective testes; however, the left vas deferens was significantly smaller in diameter. All contents within both spermatic cords appeared to have fused on the right side. A right inguinal hernia was identified and repaired using the Lichtenstein tension-free mesh technique. Postoperative course was smooth, and the patient was discharged on post-op day 1 on pain medications, to follow up with another spermogram result in the coming six months.

## Discussion

This case highlights the rare constellation of congenital genitourinary anomalies, which include the presence of a unilateral seminal vesicle agenesis and spermatic cord fusion, which is presented in the context of oligospermia and scrotal discomfort. Although each anomaly has been described in isolation, their co-occurrence presents a unique implication for fertility and surgical management [3,4].

The most exceptional finding was the fusion of both spermatic cords into a single inguinal canal. Intraoperative confirmation of two vas deferens originating from their respective testes and then traversing within the single inguinal canal helps to differentiate this case from previously discovered syndromes such as persistent Müllerian duct syndrome. 

The agenesis of the left seminal vesicle is due to the embryological disruption of the mesonephric duct, which usually forms the vas deferens, epididymis, ejaculatory ducts, and seminal vesicles. Although most cases of seminal vesicle agenesis are associated with CFTR gene mutations, as well as congenital bilateral absence of the vas deferens [1,2], this patient lacked both the CFTR gene mutation and the renal abnormality, suggesting an idiopathic cause. Unilateral seminal vesicle agenesis is frequently associated with congenital absence or malformation of the vas deferens and may be accompanied by other ipsilateral genitourinary anomalies such as renal agenesis or dysplasia due to their shared mesonephric embryologic origin. Clinically, these anomalies may present with infertility, low ejaculate volume, or scrotal pain. The American Urological Association and the American Society for Reproductive Medicine recommend imaging with transrectal ultrasound (TRUS) or MRI in men with suspected distal genital tract obstruction and abdominal imaging in those with vasal agenesis to evaluate for renal anomalies [1,2,5]. 

Varicocele, or a dilation of the pampiniform plexus, is a common abnormality that may be incidental or secondary to underlying congenital anomalies that disrupt venous drainage. In this context, the ipsilateral varicocele may further impair fertility, compounding the effects of seminal vesicle agenesis and potential vasal anomalies [6].

In summary, this clinical picture highlights the rare constellation of a congenital genitourinary anomaly. Management should be individualized, focusing on fertility assessment, imaging, and symptom relief. In cases of obstruction or congenital absence of the seminal vesicle or vas deferens, assisted reproductive technologies may be required to achieve fertility [6].

## Conclusions

This case serves to underscore the importance of a thorough patient workup, especially for males presenting with infertility and atypical scrotal anatomy. Unilateral seminal vesicle agenesis is exceedingly rare and may lead to both obstructive and/or non-obstructive subfertility. Surgical confirmation is used to affirm the presence of unusual anatomy seen on imaging. Early detection of these anomalies can help with decision management for fertility preservation, varicocele treatment, and hernia repair.
